# Family Connections versus optimised treatment-as-usual for family members of individuals with borderline personality disorder: non-randomised controlled study

**DOI:** 10.1186/s40479-017-0069-1

**Published:** 2017-08-30

**Authors:** Daniel Flynn, Mary Kells, Mary Joyce, Paul Corcoran, Sarah Herley, Catalina Suarez, Padraig Cotter, Justina Hurley, Mareike Weihrauch, John Groeger

**Affiliations:** 1 Cork Mental Health Services, Health Service Executive, Block 2, St Finbarr’s Hospital, Cork, Ireland; 2 National Suicide Research Foundation, Western Gateway Building, University College Cork, Cork, Ireland; 3 Department of Epidemiology and Public Health, Western Gateway Building, University College Cork, Cork, Ireland; 4 School of Applied Psychology, Cork Enterprise Centre, University College Cork, Cork, Ireland; 5 Nottingham Trent University, Nottingham, UK

**Keywords:** Borderline personality disorder, Family Connections, Family members, Significant others, Effectiveness, Long-term follow-up, Burden, Grief

## Abstract

**Background:**

Borderline personality disorder (BPD) is challenging for family members who are often required to fulfil multiple roles such as those of advocate, caregiver, coach and guardian. To date, two uncontrolled studies by the treatment developers suggest that Family Connections (FC) is an effective programme to support, educate and teach skills to family members of individuals with BPD. However, such studies have been limited by lack of comparison to other treatment approaches. This study aimed to compare the effectiveness of FC with an optimised treatment-as-usual (OTAU) programme for family members of individuals with BPD. A secondary aim was to introduce a long term follow-up to investigate if positive gains from the intervention would be maintained following programme completion.

**Methods:**

This study was a non-randomised controlled study, with assessment of outcomes at baseline (pre-intervention) and end of programme (post-intervention) for both FC and OTAU groups, and at follow-up (3 months post-intervention; 12 or 19 months post-intervention) for the FC group. Eighty family members participated in the FC (*n* = 51) and the OTAU (*n* = 29) programmes. Outcome measures included burden, grief, depression and mastery. Linear mixed-effects models were used to assess baseline differences in the outcome measures by gender, age group and type of relationship to the individual with BPD. Linear mixed-effects models were also used to estimate the treatment effect (FC versus OTAU) utilising all available data from baseline and end of programme.

**Results:**

The FC group showed changes indicating significant improvement with respect to all four outcome measures (*p* < 0.001). The OTAU group showed changes in the same direction as the intervention group but none of the changes were statistically significant. The intervention effect was statistically significant for total burden (including both subscales; *p* = .02 for subjective burden and *p* = .048 for objective burden) and grief (*p* = 0.013). Improvements were maintained at follow-up for FC participants.

**Conclusions:**

The findings of the current study indicate that FC results in statistically significant improvements on key measures while OTAU does not yield comparable changes. Lack of significant change on all measures for OTAU suggests that a three session psycho-education programme is of limited benefit. Further research is warranted on programme components and long-term supports for family members.

## Background

Borderline personality disorder (BPD) is a mental health diagnosis characterised by a pervasive pattern of instability of interpersonal relationships, self-image, affect, and marked impulsivity [1]. BPD typically features patterns of cognitive, emotional and behavioural dysregulation that often manifests in self-harm and suicidal behaviours [2]. Lifetime rates of approximately 69–80% for acts of self-injury, up to 75% for suicide attempts [3] and 10% for completed suicide [4] demonstrate the impact of this mental health difficulty, not just on individuals who suffer with it, but also on the family members and significant others who care for them.

Family members of individuals with BPD are often required to fulfil multiple roles such as those of advocate, caregiver, coach and guardian. It has been suggested that over time, stress can deplete family members’ capacity to cope effectively, compromising their health and life agenda [5]. Carers of those with BPD, whether related or unrelated, show higher levels of psychological and somatic distress than the general population [6]. The unpredictability of life, as a result of self-harm and suicidal behaviours [3, 4] for individuals who have a family member with a BPD diagnosis, the strain of 24-hr duty and worry, and the sense of perpetual crisis has been described as living a ‘life tiptoeing’, which can lead to feelings of powerlessness, guilt and lifelong grief [7].

Burden which has been described as “family member reported stressors due to the ill relative’s symptomatology and behaviour, both on other relationships and interfering in daily activities” [5] has been identified as higher for carers of individuals with personality disorders than for carers of those with other serious mental illnesses [8]. However, carers of individuals with BPD sometimes experience challenges and discrimination when attempting to engage with health services, are not satisfied with their involvement regarding patient discharge and support, and in general, do not feel valued, included or educated in treatment pathways [9–11]. The outlined evidence highlights the requirement of support and education, as well as relief of psychological distress for caregivers and significant others of individuals with BPD.

### Family Connections

Dialectical behaviour therapy (DBT) is one of the most researched and empirically supported interventions for treating BPD [12]. Family Connections, a programme which is based on DBT principles, was developed for relatives of individuals with BPD in an effort to meet the considerable needs of this often overlooked population [5]. The Family Connections (FC) programme is a manualised, educational, skills training and support programme which provides: current information and research on BPD and family functioning; individual coping and family skills training; and group support via shared experience with other group members [13]. The effectiveness of the FC programme for family members of individuals with a BPD diagnosis was initially explored by Hoffman et al. [5], the treatment developers, where 44 participants representing 34 families completed pre-intervention, post-intervention and 3 month follow-up self-report questionnaires. Subsequently, they also carried out a replication study with a larger population sample [14]. In both studies, the FC programme was led by family members of individuals with BPD who had trained in FC. Measures of depression, burden, grief and mastery were chosen to enable comparison with research studies undertaken on carers of those with mental illnesses other than BPD. Both studies found that participation in the FC programme led to significant reductions in grief, burden and depression and to improvements in mastery levels. In Sweden, a nine session FC programme adapted to deal specifically with suicide attempters and led by CBT therapists trained in FC, was delivered to family members of individuals who had attempted suicide [15]. A reduction in depression and burden scores was also observed.

### Current study

Previous studies on FC [5, 14] have neither included a comparison to control conditions or a follow-up longer than 3 months post-intervention. The current study was carried out in a public health setting and was limited by real world challenges such as lack of resources. An optimised treatment-as-usual (OTAU) programme was chosen as a control group. This OTAU programme consisted of a 3-week psycho-education programme for participants.

We hypothesised that the FC programme would be more effective in reducing levels of grief, burden and depression, and in increasing levels of mastery, in comparison to the OTAU programme. Secondly, we hypothesised that gains made by participants in the FC group would be maintained at long term follow-up.

## Methods

### Design and study setting

The aims of this study were to compare the 12-week Family Connections (FC) programme with a 3-week optimised treatment-as-usual (OTAU) programme, and to carry out a long term follow-up with individuals who completed the FC programme. This was a non-randomised controlled study undertaken in a public health setting in the Republic of Ireland. As there was more demand than places available for the FC programme, a 3-week psycho-education programme was offered to family members waiting for an available place on FC. This 3-week programme was the OTAU condition. Participants went directly into the FC programme if places were available; otherwise they entered the OTAU programme. Data for FC participants who first participated in the OTAU programme were not included in the analyses for the current study.

### Treatment Programmes

#### Family Connections

FC is a manualised 12 session programme, typically delivered in community settings for multiple family members/carers of individuals with BPD [13]. The FC curriculum is divided into six modules, delivered for 2 hours per session over 12 weeks and covering skills training, education on BPD, practice exercises and homework tasks. Table 1 presents an overview of the content of the six modules delivered in the FC programme.

**Table 1:** Overview of module content for FC programme

Module	Content
1	Introduction (orientation and current information on research about BPD)
2	Family Education (psycho-education on development of BPD and available treatments, transactional model)
3	Relationship Mindfulness (emotional self-management, mindfulness, letting go of judgments and decreasing emotional vulnerability)
4	Family Environment (skills to improve relationship quality; letting go of anger and acceptance skills)
5	Validation (accurate and effective self-expression, how to validate)
6	Problem Management (defining problems, collaborative problem-solving, knowing when to focus on acceptance and change)

Throughout the programme, in addition to the above outlined, participants also have time to build a support network with other FC attendees by learning together and sharing lived experiences [5].

#### Optimised treatment-as-usual

The OTAU programme was originally developed as an interim programme for family members on the waiting list for a FC programme. OTAU consisted of three didactic group sessions of psycho-education specifically oriented to family members/significant others of individuals with a BPD diagnosis. The sessions were delivered in 2 hour blocks over 3-week. Session one provided information about BPD and the DBT model. Session two gave an overview of DBT skills content so that family members had familiarity with the DBT terminology, and could understand and support their loved one. Session three focused on the importance of self-care and guidance on how to respond to a loved one with emotional and behavioural dysregulation.

As the FC programme is typically delivered by two facilitators, this format was also followed for the current study. In contrast to previous studies where trained family members were the programme facilitators [5, 14], both the FC and OTAU programmes in this study were facilitated by Clinical Psychologists, all of whom were trained in DBT and FC. The co-facilitators varied between Clinical Psychologists who were trained in DBT and FC, and family members who had completed the FC programme and FC leader training.

### Participants

Participants for the FC programme were recruited via their family member with BPD who was either attending a 12 month DBT programme, or were on the wait list for a DBT programme in their local public community mental health service. Individuals with BPD nominated one or more family members to participate in the FC programme. Participants had to be 18 years or more to participate in the intervention. Family members who wanted to participate in the FC programme then made contact with the programme facilitators to indicate their interest.

All family members who participated in the FC and OTAU programmes were invited to participate in the research study. Recruitment of participants took place from March 2011 to March 2015. All individuals who were approached consented to participate in the research evaluation resulting in 100% participation rate for this study. Eighty participants representing 53 families partook in this study. Participants ranged in age from 18 to 70 years. The majority of participants were a parent of their family member with BPD. See Table 2 for demographic information of participants.

**Table 2:** Participant demographics of the FC and OTAU groups

Variable		FC group (*n = 51*)	OTAU group (*n = 29*)
Gender	Male	23 (45%)	16 (55%)
	Female	28 (55%)	13 (45%)
Age	18–20	0 (0%)	3 (10%)
	21–30	7 (14%)	3 (10%)
	31–40	10 (20%)	2 (9%)
	41–50	11 (22%)	11 (38%)
	51–60	16 (31%)	8 (28%)
	61–70	5 (10%)	2 (7%)
	70+	2 (4%)	0 (0%)
Relative Type	Parent	29 (57%)	14 (48%)
	Spouse/Partner	14 (27%)	9 (31%)
	Other	8 (16%)	6 (21%)

### Procedure

Informed written consent was obtained by the researchers at the beginning of the study for participation in the research evaluation. Participants in both groups (FC and OTAU) then completed the outcome measures during the first session or at home if they preferred privacy for completion of measures. If completing the questionnaires at home, participants were asked to return the completed questionnaires to the researchers before the second session of the programme. The researchers were available to assist with any queries from participants if they arose.

#### Post-intervention

Participants in both groups (FC and OTAU) completed the same battery of measures in the final session of the intervention. Contact details were also obtained from participants to facilitate follow-up data collection. This was only applicable for participants who completed the FC programme. Participants who completed the OTAU programme were offered the opportunity to proceed onto the next available FC programme; therefore there was no follow-up data collection for OTAU participants.

#### Three-month follow-up

Three months after completion of the FC programme, participants were contacted via telephone to remind them that follow-up data collection was due to take place. If participants were willing to continue their participation in the research study, address details were verified so the battery of measures could be posted to them. Participants were asked to return the measures within a week of receiving them using the prepaid return envelope provided. If completed measures had not been returned during this timeframe, a text message was sent to remind participants to complete and return the measures.

#### Long-term follow-up

The procedure for data collection at long-term follow-up was the same as at 3 months post-intervention. For participants who completed the programme between 2011 and 2013, long-term follow-up was collected at 19 months following programme completion. Given resource limitations and difficulties with accessing participants at 19 month follow-up, FC participants from 2013 to 2015 were assessed at 12 month follow-up. Mann-Whitney ‘U’ tests were carried out on the data to investigate if there was a difference in scores for participants who completed measures at either the 12 or 19 month follow-up time-point. As there was no statistically significant difference between the groups, and median scores on all measures were similar for participants irrespective of whether they completed a 12 or 19 month follow-up, participants who completed either a 12 or 19 month follow-up were amalgamated into one group which is referred to as the long-term follow-up.

### Measures

In line with previous research conducted on the FC programme, the measures that were used to examine the effectiveness of both the intervention and OTAU groups were the same as those used in Hoffman et al.’s [5, 14] studies.^1^

The Burden Assessment Scale (BAS; Reinhard et al. [16]) was used to measure the construct of burden. The BAS consists of two subscales which measure objective burden and subjective burden. All items on the scale are scored to provide a total burden score. In the current study, the internal reliability of the BAS subscales and total scale ranged from .87 to .93.

The Grief Assessment Scale (GAS; Struening et al. [17]) is a 15-item scale which measures individuals’ current feelings of grief. The internal reliability of the GAS in this study was .94.

The Revised Centre for Epidemiologic Studies Depression Scale (CES-D; Radloff et al. [18]) is a 20-item scale which examines levels of depressive symptoms experienced by participants. The internal reliability of the CES-D in this study was .91.

The Personal Mastery Scale (PMS; Pearlin et al. [19]) is a 7-item self-report questionnaire which measures participants’ perceived level of coping. In the current study, the internal reliability of the PMS was .74.

### Power analysis

The study used a two-by-two repeated measures design whereby the treatment effect was based on comparing the change across time in the FC group with 51 participants to the change across time in the OTAU group with 29 participants. A posthoc power analysis was completed on the data. At the 5% level of statistical significance, assuming a correlation between pre-post measurement pairs of 0.5, a two-sided, two-sample t-test had 81% power to detect a difference in mean changes equivalent to an effect size of 0.67 standard deviations. The study had 56% and 89% power to detect effect sizes equivalent to 0.5 and 0.75 standard deviations, respectively.

### Statistical analysis

All outcome measures were quantitative and were summarised by their mean and standard deviation. Linear mixed-effects models were used to assess baseline differences in the outcome measures by gender, age group and type of relationship to the individual with BPD. Linear mixed-effects models were also used to estimate the treatment effect (FC versus OTAU) utilising all available data from baseline and end of programme. These models included a random intercept to allow for repeated measures on the same individual and also adjusted for clustering in the data as some participants came from the same family. Participant characteristics associated with baseline differences in the outcome measures were included in the models that assessed the treatment effects. Data were analysed using Stata version 13.1 for Windows.

## Results

**Fig. 1:**
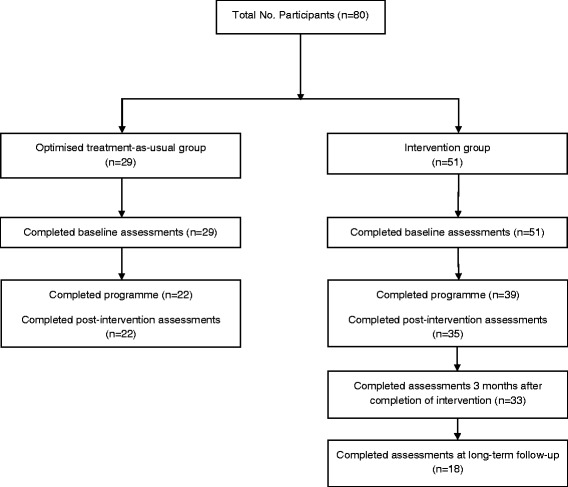
Flow of participants through the trial

Of the 80 participants originally recruited for this study, 51 participated in the FC programme and 29 in the OTAU programme (see Fig. 1). Fifty-one FC participants completed baseline assessments and 35 completed post-intervention assessments. Twelve participants did not complete the programme and four did not return measures at post-intervention. Twenty-nine OTAU participants completed baseline assessments and 22 completed post-intervention assessments. Seven participants did not complete the OTAU programme.

### Drop-out rates and missing data

Intervention drop-out rates were similar in both groups, with 24% drop-out in both the FC (12 of 51 participants) and OTAU (7 of 29 participants) groups. While all participants (22 of 22; 100%) who completed the OTAU programme completed the post-intervention outcome measures, 90% (35 of 39 participants) who completed the FC programme completed the post-intervention measures. At follow-up, 33 of 39 participants (85%) who completed the FC programme completed the 3-month follow-up measures and almost half (18 of 39; 46%) completed long-term follow-up measures.

### Baseline scores on outcome measures

Baseline scores on outcome measures for each group are reported in Table 3.

**Table 3:** Baseline scores on outcome measures for the FC and OTAU groups

Variable	FC M (SD) *n* = 51	OTAU M(SD) *n* = 29	t (df), *p*
BAS Obj	24.5 (7.3)	20.2 (5.9)	−2.7 (77), .01
BAS Sub	26.5 (7.8)	23.8 (5.6)	−1.6 (77), .11
BAS Total	51.0 (14.2)	44.0 (10.4)	−2.3 (77), .02
GAS	49.9 (14.7)	52.8 (13.4)	0.9 (78), .39
PMS	19.6 (3.5)	18.8 (4.0)	−.9 (76), .38
CESD	17.3 (10.3)	17.2 (13.0)	−.05 (73), .96

Participants in the FC group (*n* = 51) reported significantly higher scores of objective burden (BAS Obj) and total burden (BAS Total) at baseline. While FC participants also reported higher subjective burden scores (BAS Sub), this difference was not statistically significant. Although not statistically significant, participants in the OTAU group reported higher grief scores (GAS) than FC participants. Mastery (PMS) and depression scores (CESD) were similar for both groups.

### Baseline differences by participant characteristics

Gender, age and relative type were explored as constructs which could potentially impact on scores at baseline. The analyses showed that female participants had higher levels of objective burden, subjective burden, total burden, grief and depression as reported in Table 4.

**Table 4:** Effects of gender, age and relative type on baseline scores of burden, grief, mastery and depression

Variable	Ref. group	Female (vs. male)	51 years + (vs. < 51 years)	Partner (vs. parent)	Other relative (vs. parent)
	(Male, < 51 years, parent)^*^				
BAS Obj	18.8 (14.7 to 23), <0.001	+4.9 (2.3 to 7.5), <0.001	+1.8 (−2 to 5.6), 0.36	+4.9 (0.6 to 9.1), 0.025	−1.3 (−5.7 to 3), 0.549
BAS Sub	21.5 (17 to 26), <0.001	+5.3 (2.1 to 8.5), <0.001	+1.4 (−2.8 to 5.6), 0.521	+2.5 (−2.2 to 7.2), 0.298	−0.4 (−5.5 to 4.7), 0.867
BAS Total	40 (32 to 48), <0.001	+10.4 (5.2 to 15.6), <0.001	+3.4 (−4 to 10.9), 0.366	+7.6 (−0.7 to 15.9), 0.071	−2 (−10.6 to 6.6), 0.649
GAS	45 (36.4 to 53.6), <0.001	+10.6 (4.8 to 16.5), <0.001	0 (−8 to 8), 0.999	−1.3 (−10.2 to 7.6), 0.77	+0.9 (−8.5 to 10.3), 0.853
PMS	18.8 (16.6 to 21), <0.001	−0.9 (−2.6 to 0.8), 0.306	−0.1 (−2.2 to 1.9), 0.899	+1.7 (−0.6 to 4), 0.156	+3.7 (1.1 to 6.2), 0.005
CESD	10.1 (2.9 to 17.3), 0.006	+5.6 (0.4 to 10.7), 0.034	+5.2 (−1.5 to 11.8), 0.131	+5 (−2.5 to 12.5), 0.194	+1.1 (−6.9 to 9.1), 0.783

^*^estimated effect, (95% C.I.), *p* value

There were no differences according to age. When compared with parents, partners had higher levels of objective burden and other relatives reported higher levels of mastery.

### Outcome measures at pre- and post-intervention

Within the FC group, there was a significant improvement with respect to all four outcome measures (*p* < 0.001; see Table 5). Within the OTAU group, there were changes in the same direction as the intervention group but none of the changes were statistically significant. Comparing these changes between the two groups showed a statistically significant treatment effect for burden and grief.

**Table 5:** Change in outcome measures and treatment effect at post-intervention

Variable	FC group (*n* = 51) Mean change (95% CI), *p*	OTAU group (*n* = 29) Mean change (95% CI), *p*	Treatment effect Mean (95% CI), *p*
BAS Obj	−5.3 (−7.6 to −3.0), <0.001	−2.1 (−4.4 to 0.2), 0.071	−3.2 (−6.3 to 0.0), 0.048
BAS Sub	−6.3 (−8.9 to −3.6), <0.001	−1.6 (−4.1 to 0.8), 0.19	−4.7 (−8.5 to −0.8), 0.017
BAS Total	−11.6 (−16.3 to −6.9), <0.001	−3.7 (−8.2 to 0.7), 0.103	−7.9 (−14.5 to −1.2), 0.020
GAS	−9.5 (−13.6 to −5.3), <0.001	−2.1 (−6.8 to 2.6), 0.388	−7.3 (−13.1 to −1.6), 0.013
PMS	1.9 (0.8 to 2.9), <0.001	0.2 (−1.2 to 1.7), 0.739	1.6 (−0.1 to 3.4), 0.070
CESD	−5.5 (−8.6 to −2.4), <0.001	−2.0 (−6.4 to 2.4), 0.381	−3.3 (−8 to 1.4), 0.170

*Mean changes adjusted for gender and relative type*

### Outcome measures at follow-up

**Fig. 2:**
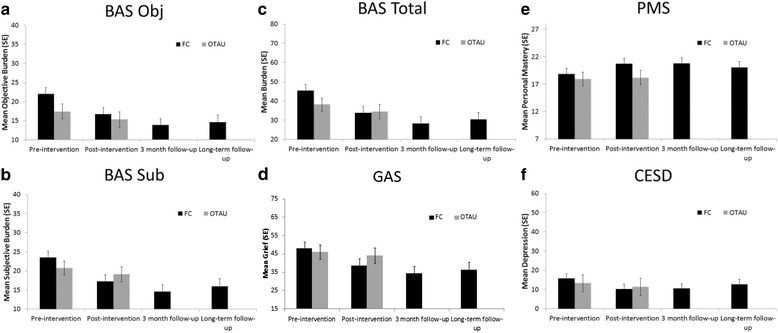
Adjusted means for participants in the FC and OTAU groups at each time-point for: **a** Objective burden; **b** Subjective burden, **c** Total burden, **d** Grief, **e** Personal Mastery, **f** Depression

During the three month period following completion of the FC programme (i.e. between T2 and T3), there were further decreases in objective burden (*p* = .026), subjective burden, total burden (*p* = .031) and grief. Depression and mastery scores showed no change at the 3 month follow-up time-point (see Fig. 2). There were no further changes observed at the long-term follow-up.

## Discussion

This was the first study to compare the effectiveness of the FC programme to a control group. The main aim of this study, to assess whether the FC programme would be more effective in reducing burden, grief, depression and in increasing levels of mastery in comparison to the OTAU group, was partially supported. A treatment effect was observed; the FC programme showed significantly larger reductions from pre- to post-intervention for burden and grief. The groups did not differ significantly in terms of change on depression or mastery scores, although the latter approached significance. This study also introduced a long term follow-up to investigate if positive gains from the intervention would be maintained at follow-up and this hypothesis was supported by the analysis.

As a relatively new programme, research to date on FC is limited; use of the measures in previous studies on FC [5, 14] helps to build the knowledge base regarding the effectiveness of FC and allowed for an examination of concordance of FC results with prior studies. Overall, results in the Irish setting were comparable to previous studies, with significant changes on all outcome measures for FC participants. This implies a consistency to the effectiveness of the FC programme independent of location.

Publicly funded health systems struggle to balance the need for an effective intervention with limited clinical resources. Research indicates that an active component of treating BPD involves structuring the environment. In structuring the environment, it is anticipated that changes among family members may be helpful to reinforce skilful behaviour of the person with BPD [20]. In the absence of staff trained in FC, the provision of psycho-education through the OTAU programme was an initial attempt to structure the environment, whereby information and understanding about BPD is provided to family members. This was offered to engage with and encourage family members to develop understanding, and thus cultivate more benign interpretations of some of the more challenging behaviours associated with BPD.

It is appreciated and acknowledged that the OTAU programme does not constitute a direct comparison group to the FC intervention due to the significant variation in duration between the programmes, and thus is not intended to be viewed as a true control. However, as a 3-week programme of short duration, the OTAU programme is a more tangible offering where there are limited resources and for the purposes of this study, gives the OTAU programme validity as a potentially viable treatment for this cohort. The 3-week OTAU programme attempted to address some of the key challenges identified for this cohort of individuals i.e. being overlooked by mental health services; managing perceived discrimination against those who care for individuals with BPD; and attempting to address inadequate support services for relatives of individuals with BPD [9, 10].

The potential impact of multiple participants representing the same individual with BPD in the groups was factored into the analysis. Hoffman et al., [5, 14] also considered this in their analysis and noted that when multiple family members participate in an intervention, their ongoing discussion of programme material and mutual support of each other’s skills practice outside of sessions may enhance their learning. This potentially impacts upon their scores on outcome variables.

The FC group in the current study differed from FC as delivered in previous research studies in the following ways: it was clinician led; participants’ family members with BPD were actively engaged in a parallel DBT programme; and participants were resident in Ireland. Nonetheless, findings on objective and subjective burden, and grief corroborate with Hoffman et al. results [5, 14]. Analyses showed that family members experienced objective and subjective burden and grief differently according to gender and type of relationship, with females and parents reporting the highest scores. Depression scores for participants in the current study were lower than those in both Hoffman et al. studies [5, 14]. It is difficult to identify a concrete reason for such disparity in scores at pre- and post-intervention; however, it is possible that cultural variance in public attitudes towards depression in an Irish context may have influenced individuals’ responses [21]. These findings have clinical implications when addressing the needs of carer well-being. Further research may assist us in understanding the challenges faced by these groups and to respond more effectively to their needs.

### Limitations

A number of limitations of the current study warrant consideration. This study took the form of a non-randomised controlled study as it would have been unethical to do random allocation when there is evidence to suggest that completion of the FC programme results in improved outcomes for participants. Additionally, at the time of this study, there was no alternative evidence-based intervention available for this participant group that could have been used as a comparison group. Therefore, no control group could be accessed for inclusion in this study. In the absence of a control group, the OTAU programme was an attempt at including a comparison group. It is acknowledged however that the discrepancy in intervention duration between the two conditions, though reflective of real world scenarios, limits the comparability between the two groups. Thus, a limitation of this study is that it uses an uncontrolled, non-randomised design, making it difficult to determine whether changes that were found were wholly due as a result of the intervention, or in part, related to other factors such as the passage of time.

The stage of participation of the individual on the DBT programme was not accounted for in the current study. Therefore it is possible that some of the changes on constructs which the current study found may have been mediated by therapeutic gains of the family member with BPD depending on what stage they were at on a DBT programme.

### Recommendations

This and previous studies attest to the effectiveness of FC. What is not known is whether it is the collective combination of the FC programme modules or one or more individual modules within the programme that are contributing to improvements in family members’ experience. Ideally, future research would allow for: an intervention and control of equal duration to allow for accurate comparisons between groups; differentiation as to whether it is intervention duration or intervention components which contribute to the construct changes; focus on control interventions of psycho-education, skills and support as individual programmes.

This study acknowledges that the FC intervention is being implemented in a scenario where the BPD diagnosed family members are concurrently undergoing DBT treatment which may be suggestive of having an additional therapeutic effect. Previous studies did not report on whether participants’ family members were engaged with a treatment programme at the time of FC participation. Future research could consider the stage of DBT treatment of the family member with BPD, and the number of family members participating in the FC programme.

Anecdotal feedback from participants indicated participant need for further skills training input following programme completion. Participants found it challenging to consolidate skills and generalise their use to other settings. This is highlighted by the lack of change in mastery scores for FC participants at follow-up. Having considered both pieces of information, we explored alternative teaching methods and tools (e.g. a DVD resource depicting family application of skills in different scenarios based on real-world examples) which might serve as a beneficial adjunct to the current manualised treatment. Additional research would be well placed to further consider whether such adjuncts could further consolidate mastery for participants who complete the FC programme.

It was earlier acknowledged that this study was different to previous studies on FC [5, 14] whereby the intervention was facilitated by clinicians rather than family members trained in FC. As the results for the most part corroborate those of previous studies, it would be of interest to examine whether or not there are in fact differences between clinician and family member led FC programmes. Prior to this study, there were no clinicians or family members trained to deliver the FC programme in Ireland. Since completion of FC training by three clinicians in 2011 and subsequent delivery of a number of programmes in Ireland, family members have both completed FC and subsequent facilitator training. Future research could consider whether clinician led or family member led FC yields the most effective results for participants.

Research to date, has with good reason, utilised a standardised battery of measures to facilitate comparison with other family studies. Future research would be well placed to critically analyse whether these measures are sufficient to extend on prior studies. For example, the construct of depression in this study was not clinically relevant for the participants in this sample. Anecdotal evidence from family members of individuals with BPD indicates that the construct of hopelessness may be of more relevance for further exploration. A qualitative exploration of participants’ experiences of the FC programme may yield information which may further guide the selection of appropriate constructs which are particularly relevant for family member of individuals with BPD.

## Conclusions

The findings of the current study indicate that FC results in statistically significant improvements on key measures while OTAU does not yield comparable changes. Lack of significant change on all measures for OTAU suggests that a three session psycho-education programme is of limited benefit. It lends support to the possibility that psycho-education only is not sufficient for change and posits the question as to whether skills training and support, the other two components of FC are the active components of change. However, given that this study did not follow a randomised design, and participant numbers for each condition were not equal with larger numbers in the FC condition, these findings should be interpreted with caution.

This is the first study of FC that has attempted to introduce a comparison group and a longer term follow-up. This also was the first study outside of the U.S., independent of the programme developers, which mirrored the published studies on the effectiveness of FC. It is important to highlight the value and benefit of system interventions, such as FC, in supporting families affected by BPD.

## Abbreviations

BAS: Burden assessment scale
BPD: Borderline personality disorder
CBT: Cognitive behaviour therapy
CES-D: Revised centre for epidemiologic studies depression scale
DBT: Dialectical behaviour therapy
FC: Family Connections
GAS: Grief assessment scale
OTAU: Optimised treatment-as-usual
PMS: Personal mastery scale

## Footnotes

1 It should be noted that the measure of mastery in the present study differed to that used in the Hoffman et al. [5, 14] studies.
